# Clinical symptoms of SARS‐CoV‐2 breakthrough infection during the Omicron period in relation to baseline immune status and booster vaccination—A prospective multicentre cohort of health professionals (SURPRISE study)

**DOI:** 10.1111/irv.13167

**Published:** 2023-06-19

**Authors:** Philipp Kohler, Baharak Babouee Flury, Sabine Güsewell, Thomas Egger, Onicio Leal, Angela Brucher, Eva Lemmenmeier, Dorette Meier Kleeb, J. Carsten Möller, Manuela Ortner, Philip Rieder, Markus Ruetti, Hans‐Ruedi Schmid, Reto Stocker, Danielle Vuichard‐Gysin, Oliver Speer, Benedikt Wiggli, Ulrike Besold, Allison McGeer, Lorenz Risch, Andrée Friedl, Matthias Schlegel, Pietro Vernazza, Christian R. Kahlert, Stefan P. Kuster, SURPRISE Study Group

**Affiliations:** ^1^ Division of Infectious Diseases and Hospital Epidemiology Cantonal Hospital St Gallen St Gallen Switzerland; ^2^ Epitrack Recife Brazil; ^3^ Department of Economics University of Zurich Zurich Switzerland; ^4^ Psychiatry Services of the Canton of St. Gallen (South) Pfäfers Switzerland; ^5^ Clienia Littenheid AG, Private Clinic for Psychiatry and Psychotherapy Littenheid Switzerland; ^6^ Division of Occupational Health Kantonsspital Baden Baden Switzerland; ^7^ Center for Neurological Rehabilitation Zihlschlacht Switzerland; ^8^ Rheintal Werdenberg Sarganserland Hospital Group Grabs Switzerland; ^9^ Hirslanden Clinic Zurich Switzerland; ^10^ Fuerstenland Toggenburg Hospital Group Wil Switzerland; ^11^ Kantonsspital Baden, Central Laboratory Baden Switzerland; ^12^ Division of Infectious Diseases and Hospital Epidemiology Thurgau Hospital Group Muensterlingen Switzerland; ^13^ Thurgau Hospital Group Institute for Laboratory Medicine Muensterlingen Switzerland; ^14^ Division of Infectious Diseases and Hospital Epidemiology Kantonsspital Baden Baden Switzerland; ^15^ Geriatric Clinic St. Gallen St. Gallen Switzerland; ^16^ Sinai Health System Toronto Canada; ^17^ Labormedizinisches Zentrum Dr Risch Ostschweiz AG Buchs Switzerland; ^18^ Private Universität im Fürstentum Liechtenstein Triesen Liechtenstein; ^19^ Center of Laboratory Medicine University Institute of Clinical Chemistry, University of Bern, Inselspital Bern Switzerland; ^20^ Department of Infectious Diseases and Hospital Epidemiology Children's Hospital of Eastern Switzerland St. Gallen Switzerland

**Keywords:** breakthrough infection, Covid‐19, health professionals, symptoms, vaccine

## Abstract

The effects of different types of pre‐existing immunity on the frequency of clinical symptoms caused by the SARS‐CoV‐2 breakthrough infection were prospectively assessed in healthcare workers during the Omicron period. Among 518 participants, hybrid immunity was associated with symptom reduction for dizziness, muscle or limb pain and headache as compared to vaccination only. Moreover, the frequencies of dizziness, cough and muscle or limb pain were lower in participants who had received a booster vaccine dose. Thus, hybrid immunity appeared to be superior in preventing specific symptoms during breakthrough infection compared to vaccination alone. A booster vaccine dose conferred additional symptom reduction.

## 1 INTRODUCTION

The protective effects of previous infection with SARS‐CoV‐2 or vaccine‐induced immunity against breakthrough infection have been significantly affected by the emergence of the Omicron variants, whereas protection against severe disease is preserved. While infection‐induced protective immunity against SARS‐CoV‐2 infection has been shown to persist for up to 1 year during the alpha and delta pandemic waves, immunity against Omicron wanes after a few months in previously infected individuals.^1^, ^2^, ^3^ Similarly, vaccine‐induced protection against Omicron infection has been shown to diminish significantly over time, and receipt of a booster vaccine dose provides only a short‐lived improvement.^2^, ^4^ As a result, the prevalence of hybrid immunity from vaccination in combination with prior or subsequent infection in the population increases progressively.^3^ Concomitantly, recent data suggest that hybrid immunity from exposures to the SARS‐CoV‐2 virus and the vaccine confers the highest level of protection from Omicron infection.^1^, ^2^, ^5^, ^6^, ^7^

Compared with the high overall burden of disease, there is a scarcity of data assessing whether individual symptoms of breakthrough infections differ between subjects with pre‐existing hybrid or vaccine‐induced immunity and whether these symptoms are affected by booster vaccine doses. In this study, we aimed to assess the effects of these different types of pre‐existing immunity on the frequency of clinical symptoms caused by the SARS‐CoV‐2 breakthrough infection during the Omicron period, with or without additional booster vaccine doses.

## 2 METHODS

### 2.1 Study design and population

The study was approved by the ethics committee of Eastern Switzerland (#2020‐00502).

Our cohort study prospectively included health care workers (HCW) 16 years of age or older from seven healthcare networks located in northern and eastern Switzerland (SURPRISE study) since June 2020. From their inclusion until March 2022, participants were followed through questionnaires on SARS‐CoV‐2 infections and corresponding symptoms, vaccinations and periodic SARS‐CoV‐2 serology measurements.^8^ Participants were asked to get tested for SARS‐CoV‐2 in case of compatible symptoms. SARS‐CoV‐2 was detected by polymerase chain reaction or rapid antigen test from nasopharyngeal swabs (NPS); self‐reported NPS results were validated as previously described.^9^ Anti‐nucleocapsid (anti‐N) and anti‐spike (anti‐S) antibodies were measured at baseline and in September 2021 using the Roche Elecsys (Roche Diagnostics, Rotkreuz, Switzerland) electro‐chemiluminescence immunoassay^10^ with an upper limit of quantification of 5000 binding antibody units (BAU)/mL.

For the current analysis, two distinct immune status groups, according to previous questionnaires and serology results as of 20 September 2021, were defined: (i) Group V (vaccinated): never reported infection and anti‐nucleocapsid (anti‐N) serum antibodies negative, but vaccinated (two doses); and (ii) Group H (hybrid immunity): reported infection or anti‐N positive (at any time) and vaccination (≥1 dose). Groups were further subdivided by either booster vaccination was received or not at least 7 days before breakthrough infection.

Our main outcomes were the symptoms reported along with the first SARS‐CoV‐2 positive NPS reported. Infections occurring at least 7 days after booster vaccination were classified as ‘preceded by booster’; those occurring within 7 days after booster vaccination were excluded. The period on and after 27 December 2021 was defined as Omicron‐dominant (i.e., the Omicron period), based on sequencing data from Eastern Switzerland^11^; only infections from this period were included in data analyses.

### 2.2 Statistical analyses

Frequencies of individual COVID symptoms were described as percentages with 95% Wilson confidence intervals. We used multivariable logistic regression to assess the impact of immune status and booster vaccination on symptom frequencies. Models included a priori selected co‐variables based on their importance in previous analyses,^12^ that is, age, sex, body mass index >30 (yes/no), any comorbidities (yes/no; diabetes mellitus, arterial hypertension, heart disease, peripheral artery disease, stroke, chronic obstructive pulmonary disease [COPD], allergic rhinitis, asthma, liver disease [fatty liver disease or cirrhosis], cancer, rheumatologic disorders [arthritis, systemic lupus erythematosus or vasculitis], inflammatory bowel disease (Crohn's disease or ulcerative colitis) or hypothyroidism) and time since last immunisation event (infection or vaccination). R version 4.0.2 was used for statistical analyses.

## 3 RESULTS

Five hundred eighteen participants had vaccination‐induced (V; 415 [80.1%]) or hybrid (H; 103 [19.9%]) immunity and developed a breakthrough infection in the Omicron period. Three hundred eighty‐two breakthrough infections (73.7%) were preceded by a booster vaccine dose. Participants in Group V were older (median age 42.0 vs. 37.4 years, *p* < 0.001) and less likely to have direct patient contact (75.6% vs. 87.6%, *p* = 0.015). There was no difference in other participants' characteristics, such as sex, body mass index, comorbidities or respirator use habits, between groups.

**FIGURE 1:**
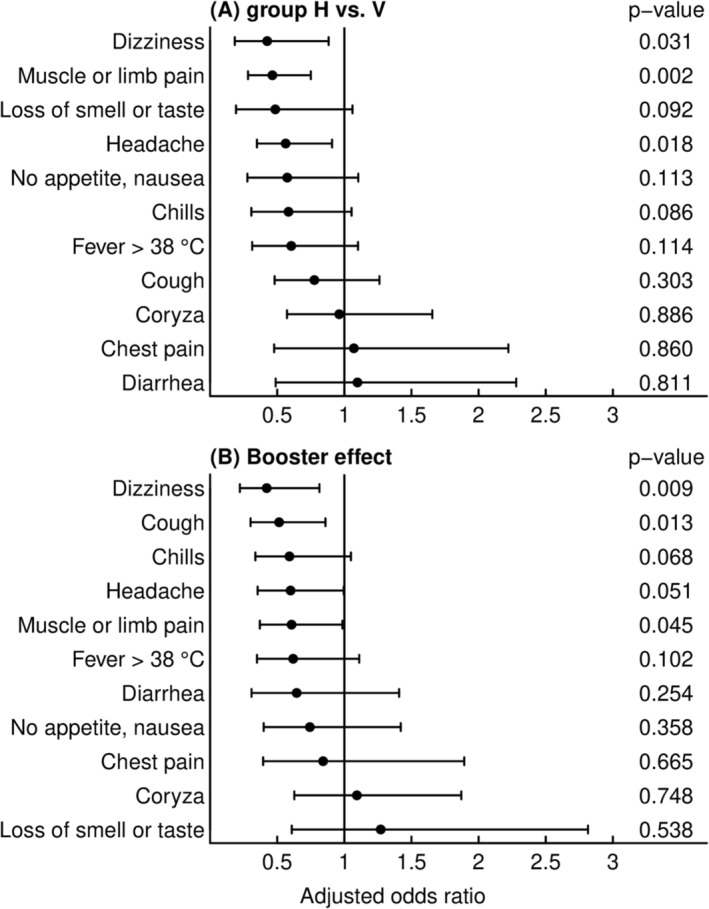
Symptoms of breakthrough infections during the Omicron period according to immune status and booster vaccination. Adjusted odds ratios (aOR) for symptoms during breakthrough infections in subjects with (A) hybrid immunity (group H) in comparison to those with vaccination (group V) only and (B) for the effect of a booster vaccine dose at least 7 days before infection during the Omicron period. Symptoms are sorted in order of increasing aOR for groups. Vertical lines (aOR = 1) indicate an absence of effect. OR shown in (A) and (B) are based on the same multivariable model and therefore adjusted for the respective other factors as well as age, sex, obesity (BMI > 30 kg/m^2^), any comorbidity and time from pre‐immunisation (i.e., either vaccination or infection prior to breakthrough infection) to breakthrough infection, which differed between those with and without booster (median 10.6 vs. 7.2 months).

The most prevalent symptoms upon breakthrough infection were coryza (76%), headache (64%), cough (64%) and muscle or limb pain (47%) (Table 1). In multivariable analyses, dizziness (adjusted odds ratio [aOR]: 0.43, 95% confidence interval [CI]: 0.18–0.88), muscle or limb pain (aOR: 0.46, 95% CI: 0.28–0.75) and headache (aOR: 0.56, 95% CI: 0.35–0.91) were less frequently reported by subjects with hybrid immunity as compared with only vaccinated individuals (Figure 1). Receipt of a booster vaccination was independently associated with reduced frequency of dizziness (aOR: 0.42, 95% CI: 0.22–0.81), cough (aOR: 0.51, 95% CI: 0.30–0.86) and muscle or limb pain (aOR: 0.61, 95% CI: 0.37–0.99) (Figure 1).

**TABLE 1:** Percentage of participants reporting each symptom (with 95% Wilson confidence intervals) for all breakthrough infections during the Omicron period and separately by group (vaccine‐induced (V) vs. hybrid (H) immunity) and receipt of any booster vaccine at least 7 days before the positive swab. Symptoms are sorted by decreasing overall frequency.

	All	Group V no booster	Group V with booster	Group H no booster	Group H with booster
	*n* = 518	*n* = 91	*n* = 324	*n* = 45	*n* = 58
Coryza	76 (72–79)	67 (57–76)	79 (74–83)	80 (66–89)	69 (56–79)
Headache	64 (60–68)	74 (64–82)	64 (58–69)	64 (50–77)	50 (38–62)
Cough	64 (59–68)	68 (58–77)	63 (57–68)	78 (64–87)	52 (39–64)
Muscle or limb pain	47 (43–52)	57 (47–67)	48 (43–54)	47 (33–61)	28 (18–40)
Chills	21 (18–25)	35 (26–45)	19 (15–23)	22 (13–36)	14 (7–25)
Fever >38°C	20 (16–23)	31 (22–41)	18 (14–22)	13 (6–26)	17 (10–29)
Loss of appetite, nausea	16 (13–19)	21 (14–30)	16 (12–20)	18 (9–31)	9 (4–19)
Dizziness	14 (11–17)	25 (17–35)	12 (9–16)	16 (8–29)	5 (2–14)
Loss of smell or taste	12 (9–15)	16 (10–25)	12 (9–16)	2 (0–12)	10 (5–21)
Chest pain	10 (7–13)	11 (6–19)	9 (6–13)	11 (5–23)	10 (5–21)
Diarrhoea	9 (7–12)	10 (5–18)	9 (6–12)	11 (5–23)	9 (4–19)

## 4 DISCUSSION

In this study, participants with hybrid immunity and those with booster vaccination experienced various symptoms of a breakthrough infection during the Omicron period less often than those who had no prior SARS‐CoV‐2 infection and no additional vaccine dose.

The major strengths of our study are the prospective observational design under real‐life conditions, the availability of serologic data allowing us to reliably determine the baseline immune status, and the collection of individual symptoms associated with SARS‐CoV‐2 infection. In suggesting a reduction in symptom frequency in subjects with hybrid immunity, our data point in the same direction as previous studies. These studies indicate better protection from infection in subjects with hybrid immunity and booster vaccination in comparison to a two‐dose vaccination regimen or infection‐based immunity only.^1^, ^2^, ^5^, ^7^ Our results are important in that they shed more light on the question of whether such differences in immune status may affect individual symptoms experienced by vaccinated individuals with breakthrough infections. Such data are particularly useful in determining whether repeated vaccinations may be of additional benefit to reduce the disease severity of SARS‐CoV‐2 breakthrough infections. While our results demonstrate that the frequency of certain symptoms was lower in boosted individuals, the impact of this finding on the risk of developing post‐COVID symptoms remains to be determined. Of note, the number of symptoms experienced during acute SARS‐CoV‐2 infection is an independent risk factor for the development of post‐COVID conditions.^12^

Our study population consists of young, mostly healthy HCW in central Europe. Therefore, our results may not be generalizable to other populations, such as children and the elderly, and those in other socioeconomic systems. Furthermore, groups were imbalanced between vaccinated participants and those with hybrid immunity. Another limitation is that SARS‐CoV‐2 testing was not mandatory, that the results of the NPS and symptoms associated with SARS‐CoV‐2 infection were self‐reported and not confirmed by other tests within the study setting, and that our validation of self‐reported NPS was done prior to the Omicron period. Nevertheless, the possibility to match the self‐reported infections with symptoms in chronological correlation strengthen our results.

Furthermore, we did not assess the severity and duration of symptoms and whether the reduction of symptoms led to less work absenteeism. Last, we cannot exclude that some individuals from the vaccinated but purportedly uninfected group (Group V) had asymptomatic infections prior to the symptomatic episode, which should have led to their assignment to the hybrid immunity group (Group H). This may have biassed the estimate to the null. Nevertheless, the fact that a difference could be shown strengthens our findings that a true difference exists.

In conclusion, hybrid immunity and the receipt of a booster vaccine dose were associated with a reduction of a number of symptoms from SARS‐CoV‐2 breakthrough infections during the Omicron wave. Further studies are needed to determine whether there is an effect on the development of post‐COVID conditions and if such symptom reductions vary depending on future variant‐adapted vaccine boosters.

## AUTHOR CONTRIBUTIONS

Philipp Kohler, Christian R. Kahlert, Allison McGeer and Stefan P. Kuster were involved in the conception and design of the work; Thomas Egger, Onicio Leal, Angela Brucher, Eva Lemmenmeier, Dorette Meier Kleeb, J. Carsten Möller, Manuela Ortner, Philip Rieder, Markus Ruetti, Hans‐Ruedi Schmid, Reto Stocker, Danielle Vuichard‐Gysin, Benedikt Wiggli, Ulrike Besold and Andrée Friedl were involved in data acquisition; Philipp Kohler, Baharak Babouee Flury, Sabine Güsewell, Matthias Schlegel, Christian R. Kahlert and Stefan P. Kuster interpreted the data; Sabine Güsewell performed the statistical analyses; Oliver Speer and Lorenz Risch were responsible for serology measurements; Philipp Kohler, Pietro Vernazza, Christian R. Kahlert and Stefan P. Kuster were responsible for study funding; Stefan P. Kuster drafted the original version of the manuscript. All authors read and approved the final manuscript.

## CONFLICT OF INTEREST STATEMENT

No conflict of interest was declared.

### PEER REVIEW

The peer review history for this article is available at https://www.webofscience.com/api/gateway/wos/peer-review/10.1111/irv.13167.

## Data Availability

The data presented in this manuscript have not been made publicly available.
